# Prevalence of HBV and HCV infections, Bhutan, 2017: Progress and next steps

**DOI:** 10.1186/s12879-020-05176-3

**Published:** 2020-07-08

**Authors:** N. Tshering, G. P. Dhakal, U. Wangchuk, S. Wangdi, L. Khandu, S. Pelden, F. Nogareda, M. K. Patel, Y. J. F. Hutin, K. Wannemuehler, B. B. Rewari, S. Wangchuk

**Affiliations:** 1 Department of Public Health, Ministry of Health, Thimphu, Bhutan; 2 Department of Medicine, Jigme Dorji Wangchuk National Referral Hospital, Thimphu, Bhutan; 3 WHO Country Office, Thimphu, Bhutan; 4 Royal Centre for Disease Control, Department of Public Health, Ministry of Health, Thimphu, Bhutan; 5 WHO Headquarters, 20 Avenue Appia, 1211 Geneva, Switzerland; 6 WHO Regional Office for the Eastern Mediterranean Region, Monazamet El Seha El Alamia Street, Nasr City, Po Box 7608, Cairo, 11371 Egypt; 7 United States Centers for Disease Control and Prevention, Atlanta, GA USA; 8 WHO Regional Office for the South East Asia Region, New Delhi, India

**Keywords:** Hepatitis B, Epidemiology, Immunization, Prevention of mother to child transmission, Perinatal infections, Evaluation, Survey HBsAg

## Abstract

**Background:**

Bhutan is committed to eliminating hepatitis B and hepatitis C, though recent baseline estimates of disease burden in the general population are unknown. In 2017, we carried out a biomarker survey in the general population to estimate the prevalence of hepatitis B virus (HBV) and hepatitis C virus (HCV) biomarkers to evaluate the impact of immunization and guide further efforts.

**Methods:**

In 2017, a cross-sectional, population-based, three-stage cluster survey was undertaken of the general population (1–17 and 20+ years of age). We visited households, collected blood specimens and administered a standard questionnaire. Specimens were collected for hepatitis B surface antigen (HBsAg) and hepatitis C virus antibody (anti-HCV) testing. We calculated prevalence of infection and selected characteristics, along with confidence intervals (CIs).

**Results:**

Of 1372 individuals approached, 1358 (99%) participated. Of those, 1321 (97%) had a specimen tested for HBsAg, and among 1173 enrolled individuals 5 years of age or older, 1150 (98%) individuals were tested for anti-HCV. The prevalence of HBsAg was 2.0% in 775 persons 20 years of age or older (95% CI: 1.0–4.0) and 0.5% in 546 persons 1–17 years of age (95% CI: 0.1–1.8). The prevalence of anti-HCV was 0.3% (95% CI: 0.1–0.8) among persons ≥5 years.

**Conclusions:**

Universal hepatitis B immunization of infants has resulted in a low prevalence of chronic HBV infection in persons 1–17 years of age and the prevalence of anti-HCV is low among persons aged ≥5 years. Efforts should continue to reach high coverage of the timely birth dose along with completion of the hepatitis B vaccine series. To reduce the chronic liver disease burden among adults, HBV and HCV testing and treatment as indicated might be restricted to pregnant women, blood donors, individuals with chronic liver diseases, and other groups with history of high-risk exposures.

## Background

WHO estimates that in 2015, viral hepatitis led to 1.34 million deaths worldwide [1]. Sequelae of chronic infections with hepatitis B virus (HBV) and hepatitis C virus (HCV) accounted for more than 90% of viral hepatitis mortality [1]. In 2016, the World Health Assembly (WHA) approved the first Global Health Sector Strategy (GHSS) on viral hepatitis [2]. The GHSS on viral hepatitis calls for elimination of viral hepatitis as a public health threat by 2030, defined as reducing incidence by 90% and mortality by 65%. By 2020, the GHSS also proposes to reach ≤1% prevalence of chronic HBV infection among children 5 years of age. In 2016, the South East Asia Region’s Immunization Technical Advisory Group established a regional goal to achieve ≤1% prevalence of hepatitis B surface antigen (HBsAg) among 5-year-old children by 2020 (http://www.searo.who.int/immunization/documents/sear_itag_2016.pdf?ua=1). To formulate action plans for elimination, burden of disease estimates are needed. Since most chronic HBV and HCV infections are asymptomatic [3], biomarker surveys in the general population are necessary [4]. In the WHO South East Asia Region, countries have started to conduct initial assessments to inform and prioritize national strategies.

The Kingdom of Bhutan had an estimated population of 807,000 in 2017 (United Nations population estimates). In 1995–96, a serological survey indicated that 5.9% of the general population were HBsAg positive [5, 6] (intermediate endemicity for chronic HBV infection, i.e., 2–8% prevalence of HBsAg [7]). In 1997, a three-dose hepatitis B vaccine schedule was introduced into the Expanded Programme on Immunization [EPI]. From 2000 and 2004 onwards, coverage stabilized above 80 and 90%, respectively. In 2012, a hepatitis B vaccine birth dose was added to the childhood immunization schedule. Birth dose coverage was 29% in 2011, increased to about 60% in 2012–2013, and further increased to 82% in 2016, based on administrative reports.

In 2016, anecdotal reports from clinicians identified a large number of adult patients with chronic liver disease in health care facilities (Guru Prasad Dhakal, Jigme Dorji Wangchuk National Referral Hospital, Thimphu, Bhutan, personal communication); a high proportion of these patients were too old to have received hepatitis B vaccination and had chronic HBV infection. However, the prevalence of hepatitis viral infections among adults in the general population was unknown, since no biomarker surveys have been conducted since 1997. Moreover, a population-based biomarker survey among children was needed to document progress towards reaching the regional hepatitis B control goal. Therefore, a cross-sectional biomarker sero-survey was conducted to estimate the prevalence of HBV and HCV infections among those born before 1997 and after 2000, in order to (1) evaluate the impact of hepatitis B vaccination on the burden of chronic HBV infection in children and (2) quantify the burden of chronic hepatitis B and C virus infections in adults. The findings were used to inform development of a national viral hepatitis plan.

## Methods

### Design

The design was a cross-sectional, population-based, three-stage cluster survey. We integrated the survey with a measles-rubella serological survey to reduce cost and improve efficiencies. This report describes the hepatitis B and C results.

### Population

The survey population consisted of household members living in Bhutan at the time of the survey. Three age groups were defined: 1) persons born from 1 April 2012 to 31 March 2016 who were aged 1–4 years (‘young children’) at the time of the survey; 2) persons born from 1 April 2000 to 31 March 2012 who were aged 5–17 years (‘older children’) at the time of the survey. 1 April 2000 was chosen to include children who had had an opportunity to receive three doses of hepatitis B vaccine after the vaccine was introduced in 1997, excluding period from 1997 to 1999 when coverage was low); and 3) persons born before 1997 who were aged ≥20 years (‘adults’) at the time of the survey.

### Operational definitions

We defined chronic HBV infection as the presence of HBsAg, past or present HCV infection as the presence of HCV antibodies, and chronic HCV infection as the presence of HCV RNA.

### Sampling

#### Sample size

To address the primary objective of the survey (estimating the prevalence of HBsAg among adults and older children), the sample size was estimated on the basis of the expected prevalence of the biomarker in the surveyed population, the absolute precision, and the intraclass cluster correlation. For younger and older children together, we expected a prevalence of 1% (the regional control goal threshold), an absolute precision of 1% and a design effect of 1.5. This generated a sample size of 571 children, using the asymptotic formula, increased to 657 children after adjustment for an anticipated 15% non-response. For adults, we expected a HBsAg prevalence of 5% (the prevalence before hepatitis B vaccine introduction), an absolute precision of 2% and a design effect of 1.5. This generated a sample size of 685 adults, increased to 788 after adjustment for an anticipated 15% non-response. For younger children aged 1–4 years, we also calculated a minimum effective sample size based on the sample size needed for the concomitant measles-rubella serosurvey. Sample size was calculated based on the assumption of an expected 95% seroprevalence of measles immunoglobulin (IgG), a desired precision of a one-sided confidence interval of − 5% and a significance level α = 0.05, which resulted in an effective sample size of 123 (Fleiss formula). Accounting for the cluster design with a target of eight young children per primary sampling unit, an intraclass correlation = 0.1, and an anticipated 15% nonresponse, the sample size was increased to 245 young children. A design of 30 clusters with 29 households per cluster was chosen to achieve the minimum sample size in each age group.

#### Cluster sampling

The sample was stratified to separate Thimphu, the capital, from the rest of the country, in order not to oversample in the most populous area of the country. In the first stage, four gewogs (i.e., blocks of villages, with population size around 4000) were chosen from Thimphu and 26 from the rest of country, by probability proportional to estimated size with replacement from a national census list. In the second stage, an updated household line list was created from which 29 households were randomly selected and visited. At each household, field workers listed those eligible individuals (anyone who had been living in the household for the last 6 months, regardless of presence at home during the first visit). One participant was selected from each of the three age groups. If there was more than one eligible individual in an age group, one was selected at random using a random number generated by a cell phone application (third sampling stage). In case of absences, field workers re-visited up to three times on two different days. If the selected individual remained absent after 2 days, the individual was counted as absent and not replaced.

### Data collection

Trained field workers administered one of three standardized questionnaires (based on the age group) to the enrolled individuals or their parents after obtaining written consent (and assent as appropriate). The questionnaire included demographic characteristics (e.g., age, gender, residence); hepatitis B vaccination history; each participant’s or caregiver’s knowledge of HBV or HCV infection status; and exposures that could be risk factors for HBV or HCV infection. Vaccination data were verified by reviewing vaccination cards in those 1–17 years of age when available. Questions were asked in a private setting to ensure confidentiality. Pre-test counselling was conducted as per the WHO guidelines on viral hepatitis testing. Written consent was requested for individuals in all three age groups for hepatitis B testing and among older children and adults for hepatitis C testing. Venous blood was collected from consenting selected individuals following standard quality control. Plasma was separated at the nearest Basic Health Unit (BHU), stored at -20 °C, and sent to Royal Centre for Disease Control (RCDC) under temperature control for testing.

### Laboratory testing

At RCDC, quality assurance and quality control in specimen processing were carried out using standard operating procedures of the laboratory. The survey specimens were tested together with quality control specimens supplied by the National Reference Laboratory, Melbourne, Australia [8]. HBsAg testing was performed using the MUREX HBsAg Version 3 (DiaSorin UK, United Kingdom); and for HCV, anti-HCV antibody testing was conducted using the INNOTEST HCV Ab IV (Fujirebio Europe, Belgium). Participants who were HBsAg positive who could be followed up were tested for quantitative HBV DNA, and those who were anti-HCV positive were tested for HCV RNA at an ISO 9001-2000 certified, College of American Pathologists accredited clinical laboratory in New Delhi, India on a second specimen.

### Return of tests results to participants

After laboratory testing was completed, investigators returned the results of the hepatitis B and C tests to the participants, if positive, and provided post-test counselling according to WHO guidelines on viral hepatitis testing [6]. Investigators organized referral for further assessment, care and treatment for those testing HBsAg or anti-HCV positive.

### Analysis plan

Data were double entered using Epi Info™ v7 . Weights were applied, taking into account the sampling strategy, non-response, and the population distribution in Bhutan. Concerning vaccination, timely hepatitis B birth dose (HepB-BD) was defined as a dose given on the day of birth or on the day after birth. Frequencies, weighted percentages, and 95% logit confidence intervals were calculated for all items. We compared populations with and without selected characteristics in terms of prevalence of biomarkers through the calculation of prevalence ratios and their 95% CIs.

### Human subjects protection

The Research & Ethical Board of Health, Bhutan, and the WHO Research Ethics Review Committee approved the survey protocol after the necessary amendments (Bhutan: REBH/Approval/2017/008; WHO: ERC.0002874). This activity was reviewed in accordance with CDC human research protections procedures and was determined to be nonresearch, programme evaluation.

## Results

### Recruitment of participants

In March–April 2017, 870 households were visited in 30 clusters in 13 districts (Dzongkhags). Among them, 781 (90%) households agreed to participate in the survey with at least one member of the household, 18 (2%) households refused to participate, and 71 (8%) were ineligible (non-residential, abandoned, or no adults available). From the 781 participating households, 1372 individuals were approached for participation. Of those, 1358 (99%) individuals were interviewed (779 adults aged ≥20 years, 394 children aged 5–17 years and 185 children aged 1–4 years); 1321 (97%) blood specimens were collected and tested for HBsAg; and specimens from 1150 (98%) of 1173 older children and adults were tested for anti-HCV.

### Chronic HBV infection prevalence

Of the 775 adults tested (median age: 41 years, inter-quartile range: 32–53), 15 (2%, 95% CI 1–4%) were HBsAg positive (Table 1). Of the 375 older children tested, three (0.7%, 95% CI 0.2–2.3%) were HBsAg positive. None of the 171 young children tested were HBsAg positive. Overall, of the 18 persons who were HBsAg positive, only one was aware of their infection. HBsAg prevalence was 1.8% in rural areas (95% CI: 0.8–4.1%) and 0.7% in urban areas (95% CI: 0.2–2.3%). All 18 HBsAg-positive persons were born in Bhutan.

**Table 1:** Prevalence of chronic HBV infection (HBsAg positive) and serological evidence of past or present HCV infection (anti-HCV positive) by gender, age, residence and country of birth, Bhutan, 2017

		HBsAg			Anti-HCV		
		Tested	Positive	% [95% CI]	Tested	Positive	% [95% CI]
**Gender**	**Female**	693	7	1.3 [0.5–3.5]	603	3	0.3 [0.1–1.0]
	**Male**	627	11	1.9 [0.9–3.7]	547	1	0.2 [0.0–1.9]
**Age**	**1–4 years**	171	0	0 [0–2.1] ^a^	0	–	–
	**5–17 years**	375	3	0.7 [0.2–2.3]	375	1	0.5 [0.1–3.4]
	**1–17 years**	546	3	0.5 [0.1–1.8]	0	–	–
	**≥20 years**	775	15	2.0 [1.0–4.0]	775	3	0.2 [0.1–0.7]
**Residence**	**Rural**	1071	16	1.8 [0.8–4.1]	928	4	0.4 [0.1–1.1]
	**Town**	250	2	0.7 [0.2–2.3]	222	0	–
**Country of birth**	**Bhutan**	1312	18	1.6 [0.8–3.2]	1141	4	0.3 [0.1–0.8]
	**India**	7	0	–	7	0	–
**Total**		**1321**	**18**	**1.6 [0.8–3.2]**	**1150**	**4**	**0.3 [0.1–0.8]**

^a^Exact confidence interval

#### Factors associated with chronic HBV infection

The prevalence of HBsAg did not vary according to the exposures examined, including traditional or formal health care occupation, history of blood transfusion, or invasive treatment by traditional healers (Table 2).

**Table 2:** Prevalence of chronic HBV infection (HBsAg positive) and serological evidence of past or present HCV infection (anti-HCV positive) according to selected characteristics, Bhutan, 2017

Outcome	Past exposure to formal or traditional health care	Prevalence among persons with the characteristic			Prevalence among persons without the characteristic			Prevalence Ratio [95% CI]
		N	Positive	% [95% CI]	N	Positive	% [95% CI]	
**Chronic HBV infection (HBsAg-positive)**	**Village health worker**	13	0	–	762	15	2.0 [1.0–4.1]	–
	**Health care worker**	13	0	–	762	15	2.0 [1.0–4.1]	–
	**Traditional healer**	5	0	–	764	15	2.0 [1.0–4.1]	–
	**Blood transfusion**	59	1	1.7 [0.2–12.3]	713	14	2.0 [1.0–4.3]	0.8 [0.1–7.8]
	**Invasive treatment by healer**	156	6	3.7 [1.6–8.3]	550	7	1.4 [0.5–3.7]	2.6 [0.7–9.6]
**Serological evidence of past or present HCV infection (anti-HCV)**	**Village health worker**	13	0	–	762	3	0.2 [0.1–0.7]	–
	**Health care worker**	13	0	–	762	3	0.2 [0.1–0.7]	–
	**Traditional healer**	5	0	–	764	3	0.2 [0.1–0.7]	–
	**Blood transfusion**	59	0	–	713	3	0.2 [0.1–0.8]	–
	**Invasive treatment by healer**	156	2	0.9 [0.2–3.7]	550	1	0.1 [0.0–0.6]	12.6 [1.0–160.0]

Among young children, vaccination coverage was higher for the third dose (92%) of the routine series than for the birth dose (70%) (Table 3). Among older children, who were born before the introduction of the HepB-BD, coverage was much lower both for the HepB-BD (16%) and for the third dose (59%), than among young children (Table 3). The low prevalence of HBsAg, small sample size, and moderate-to-high vaccination coverage prevented comparisons between vaccination status and HBsAg status, as confidence intervals were too wide (Table 3).

**Table 3:** Vaccination coverage and prevalence of chronic HBV infection (HBsAg positive) according to vaccination status, Bhutan, 2017

					Prevalence of HBsAg among 171 children 1-4 years and 375 children 5-17 years with test performed and available			
		Weighted average of vaccination coverage			Among vaccinated		Among unvaccinated	
		Vaccinated	Total	(%)	N/Total (%)	95% confidence interval	N/Total (%)	95% confidence interval
**Children 1–4 years**^a^	**Birth dose**^b^	136	185	70%	0/127 (0%)	–	0/44 (0%)	–
	**Third dose**	174	185	92%	0/164 (0%)	–	0/7 (0%)	–
	**Three doses, with birth dose**	135	185	70%	0/126 (0%)	–	0/45 (0%	–
**Children 5–17 years**^c^	**Birth dose**^d^	45	394	16%	1/40 (2.4%)	0.4–15.0	2/335 (0.4%)	0.1–1.5
	**Third dose**	253	394	59%	3/246 (1.1%)	0.3–3.9	0/129 (0%)	–

^a^171 specimens tested

^b^Any birth dose, 128/136 given within 24 h of birth

^c^375 specimens tested

^d^Any birth dose, 38/45 given within 24 h of birth

### HCV infection prevalence

Of the 1150 older children and adults tested, four were positive for anti-HCV (0.3%, 95% CI: 0.1–0.8%, Table 1). Prevalence did not significantly vary with age; 0.5% (one of 375, 95%CI: 0.1–3.4%) among older children and 0.2% (three of 775, 95%CI: 0.1–0.7%) among adults. All four persons who were anti-HCV positive lived in rural areas (0.4%, 95% CI: 0.1–1.1%) and were born in Bhutan; none were aware of their anti-HCV positive status.

#### Factors associated with HCV infection

Anti-HCV prevalence did not vary according to traditional or formal health care occupation or history of blood transfusion (Table 2). Anti-HCV prevalence was higher among those with a history of invasive treatment by traditional healers (0.9%) than among persons without such a history (0.1%), but the 95% confidence interval of the prevalence ratio included one (Table 2).

### Referral to care

#### Referral to care among HBsAg positive persons

Of the 18 persons who were identified to be HBsAg positive, 12 (67%) could be referred to care (others were lost to follow up). Of those, three (25%) were HBV DNA positive. One HBV DNA positive person had an increased alanine amino transferase level, and was eligible for treatment according to 2015 WHO HBV treatment guidelines; this individual began Tenofovir treatment.

#### Referral to care among anti-HCV positive persons

Of the four persons who were identified to be anti-HCV positive, none were HCV RNA positive. None of the anti-HCV positive persons was eligible for treatment according to the 2018 WHO HCV treatment guidelines.

## Discussion

One of the purposes of this biomarker survey was to evaluate the impact of hepatitis B vaccination among children. In 2016, the Immunization Technical Advisory Group of the WHO South East Asia Region set a goal to reduce the HBsAg prevalence among 5 year old children to < 1% by 2020, in agreement with the goal of the GHSS on Viral Hepatitis [2]. In this study, children aged 5–17 years had a HBsAg prevalence of 0.7%, suggesting that the regional goal might have been already achieved, but the wide confidence limits (range: 0.2–2.3%) prevent a definitive conclusion. In this survey, 92% of 1–4 year old children had received three hepatitis B vaccine doses, and 70% had received three doses and a birth dose; however, only 59% of the 5–17 years of age had received three hepatitis B vaccine doses. These findings are consistent with hepatitis B vaccination coverage from administrative reports. Pre-vaccine HBsAg seroprevalence was estimated to be 5.2% among children 0–12 years of age [5], which represents an 86% reduction in the prevalence of chronic HBV infection. This reduced prevalence in children is expected to reduce morbidity and mortality from HBV-related chronic liver diseases in the coming years [7]. In addition, when girls who were vaccinated as infants reach childbearing age, Bhutan will benefit from a second-generation effect of decreased mother-to-child transmission that will further reduce the incidence of chronic HBV infections in children 5 years of age [9]. Chronic HBV infections in children 5 years of age is the indicator of the ‘combatting hepatitis’ target of the sustainable development goal [10]. In 2019, the South-East Asia Region Expert Panel for Verification of hepatitis B control [11] considered the length of the nationwide vaccination programme, schedule, coverage and HBsAg prevalence in this survey and concluded that the country had achieved the hepatitis B control target of HBsAg 1% in children at least 5 years old. The Regional Expert Panel underlined that while a point prevalence of < 1% was obtained, the 95% confidence interval was larger than desired, thus alone not allowing a definite conclusion. However, evidence from the vaccination programme, including long history of hepatitis B vaccination, the high, sustained coverage in the last decade and the inclusion of a birth dose in 2011 added confidence to the finding of < 1% HBsAg prevalence in children 5–17 years old.

This biomarker survey was also an opportunity to evaluate the HBsAg prevalence among adults in the context of developing a strategy to identify and manage persons with chronic HBV infection. Overall, among persons 20 years of age or older, HBsAg the prevalence was 2%, lower than in the 1995 survey (6.3% among persons 24 years of age or older) [5]. Projections of this prevalence to the adult population in Bhutan (about 500,000 persons) suggest an estimated 5000 to 20,000 persons living with chronic HBV infection in the country in 2017. Vaccination cannot explain the reduction of HBsAg in adults during 1995–2017, since this age cohort was born before hepatitis B vaccine was introduced in the routine childhood immunization schedule. One possible explanation for the reduction in HBsAg prevalence would be that endemicity started to decrease because of improved infection control practices before immunization [12]. Another possible explanation is that older age groups might have suffered increased mortality from HBV infection [13], and the increased mortality would have reduced the prevalence between 1995 and 2017. With the relatively low prevalence among adults, a focused testing and treatment strategy might be considered in Bhutan (e.g., testing pregnant women, individuals with chronic liver diseases, populations with history of high-risk exposures, and blood donors) [6]. WHO recommends routine testing for all adults only if the HBsAg prevalence exceeds 2–5% in the general population [6].

Overall, the prevalence of anti-HCV was lower (0.3%) than in the 1995 biomarker survey (1.3%). Projections of the HCV infection prevalence to the adult population in Bhutan, and assuming 80% of anti-HCV positive persons are HCV RNA positive (the proportion estimated for India [14]) suggest an estimated 400 to 2800 persons living with chronic HCV infection in the country in 2017. Two factors could explain the lower prevalence in 2017 than in 1995. First, the serological assay that we used in 2017 may have had a higher sensitivity and specificity than the Abbott ELISA assay used in 1995, an earlier generation test that was less sensitive and less specific than the current ones [15–17]. Seroprevalence studies done before 2000 with first and second generation anti-HCV tests had too many false positive [14]. Second, under the assumption that transmission took place on a larger scale in the past, some older individuals with HCV infection may have died from chronic liver disease in the meantime, which would have led to a reduction of prevalence. In many countries, HCV transmission was more intense at the end of the twentieth century than it has been in the twenty-first century [14, 18].

Our survey also provided an opportunity to examine prevalence of HCV infection according to histories of possible exposures. Cross sectional surveys that examine serological evidence of past or present HCV infection are not a good way to explore the modes of transmission of viruses like HCV that lead to chronic infection. However, results of our analysis suggest that the prevalence of anti-HCV was higher among persons who had received invasive procedures from traditional healers. This finding is consistent with what is known of the modes of transmission of HCV [19]. Given the low prevalence of chronic HCV infection in Bhutan, a focused testing and treatment strategy might be considered for individuals with chronic liver diseases, populations with history of high-risk exposures, and blood donors [6]. General population testing may not be appropriate as WHO recommends routine testing for HCV infection for adults only if the HCV infection prevalence exceeds 2–5% in the general population [6].

Our survey had a number of limitations. First, some questions relied on recall data, such as history of exposure to invasive procedures, some of which might have occurred long ago, and vaccination status of some children. Secondly, while participation was high, some selected households did not participate, and it is unknown if these households were different from participating households. The subsets of individuals with exposures of interest were quite small; therefore, we might have failed to find significant associations with infection, even if they existed. Overall, our small sample size and low prevalence did not allow us to identify population subgroups that should be tested for HBV or HCV infection. Thirdly, expected differences in HBsAg prevalence estimates between age categories may have been diluted, as older children also had received birth dose while some adults could have been vaccinated as young adults. Fourth, the low proportion of HBsAg and anti-HCV positive persons who were positive for HBV DNA (3/1) and HCV RNA (0/4) may point to a need to confirm positive serological tests with a second test. Finally, we did not examine children born when hepatitis B vaccine was first introduced in the routine childhood vaccination schedule (i.e., from 1997 to 1999), preventing us from inferring the prevalence estimate found in this study directly to the entire population of Bhutan.

## Conclusions

In conclusion, our survey documented that the prevalence of HBV infection is likely to be lower than 1% in the children 5–17 years of age, probably because of moderate-to-high vaccination coverage. We also found that the prevalence of HBV infection in the total population was under the threshold of 2% (low endemicity) while the past or present HCV infection prevalence was very low (0.3%). Based on these results, efforts should continue to reach high coverage of the timely birth dose along with completion of the hepatitis B vaccine series [20]. For adults, HBV and HCV testing might reasonably be restricted to pregnant women, blood donors, individuals with chronic liver diseases, and other groups with history of high-risk exposures such as injection drug use for HCV infection.

## Data Availability

The datasets used and/or analysed during the current study are available from the corresponding author on reasonable request.

## Abbreviations

Anti-HCV: Hepatitis C virus antibody
CI: Confidence Intervals
HBV: Hepatitis B virus
HCV: Hepatitis C virus
HBsAg: Hepatitis B surface antigen
HepB-BD: Hepatitis B birth dose
IgG: Immunoglobulin G
RCDC: Royal Centre for Disease Control
